# Potential prognostic markers and significant lncRNA–mRNA co-expression pairs in laryngeal squamous cell carcinoma

**DOI:** 10.1515/biol-2021-0052

**Published:** 2021-06-02

**Authors:** Junguo Wang, Dingding Liu, Yajun Gu, Han Zhou, Hui Li, Xiaohui Shen, Xiaoyun Qian

**Affiliations:** Department of Otolaryngology Head and Neck Surgery, Affiliated Drum Tower Hospital of Nanjing University Medical School, Jiangsu Provincial Key Medical Discipline (Laboratory), No. 321 Zhongshan Road, Nanjing, 210008, China; Department of Otolaryngology, Research Institute of Otolaryngology, No. 321 Zhongshan Road, Nanjing, 210008, China; Research Institute of Otolaryngology, No. 321 Zhongshan Road, Nanjing, 210008, China

**Keywords:** laryngeal squamous cell carcinoma, differential expression, co-expression analysis, lncRNA–mRNA, prognosis

## Abstract

lncRNA–mRNA co-expression pairs and prognostic markers related to the development of laryngeal squamous cell carcinoma (LSCC) were investigated. The lncRNA and mRNA expression data of LSCC in GSE84957 and RNA-seq data of 112 LSCC samples from TCGA database were used. Differentially expressed genes (DEGs) and lncRNAs (DE-lncRNAs) between LSCC and para-cancer tissues were identified. Co-expression analysis of DEGs and DE-lncRNA was conducted. Protein–protein interaction network for co-expressed DEGs of top 25 DE-lncRNA was constructed, followed by survival analysis for key nodes in co-expression network. Finally, expressions of several DE-lncRNAs and DEGs were verified using qRT-PCR. The lncRNA–mRNA network showed that ANKRD20A5P, C21orf15, CYP4F35P, LOC_I2_011146, XLOC_006053, XLOC_I2_003881, and LOC100506027 were highlighted in network. Some DEGs, including FUT7, PADI1, PPL, ARHGAP40, MUC21, and CEACAM1, were co-expressed with above lncRNAs. Survival analysis showed that PLOD1, GLT25D1, and KIF22 were significantly associated with prognosis. qRT-PCR results showed that the expressions of MUC21, CEACAM1, FUT7, PADI1, PPL, ARHGAP40, ANKRD20A5P, C21orf15, CYP4F35P, XLOC_I2_003881, LOC_I2_011146, and XLOC_006053 were downregulated, whereas the expression of LOC100506027 was upregulated in LSCC tissues. PLOD1, GLT25D1, and KIF22 may be potential prognostic markers in the development of LSCC. C21orf15-MUC21/CEACAM1/FUT7/PADI1/PPL/ARHGAP40 are potential lncRNA–mRNA pairs that play significant roles in the development of LSCC.

## Graphical abstract

Several genes, PLOD1, GLT25D1, and KIF22, are identified as potential prognostic markers for LSCC development. In addition, lncRNA–mRNA pairs related to LSCC were explored, and 6 DEGs (MUC21, CEACAM1, FUT7, PADI1, PPL, and ARHGAP40) and 7 DE-lncRNAs (ANKRD20A5P, C21orf15, CYP4F35P, XLOC_I2_003881, XLOC_I2_011146, XLOC_006053, and LOC100506027) were validated to be abnormally expressed in LSCC tissue.

**Figure:**
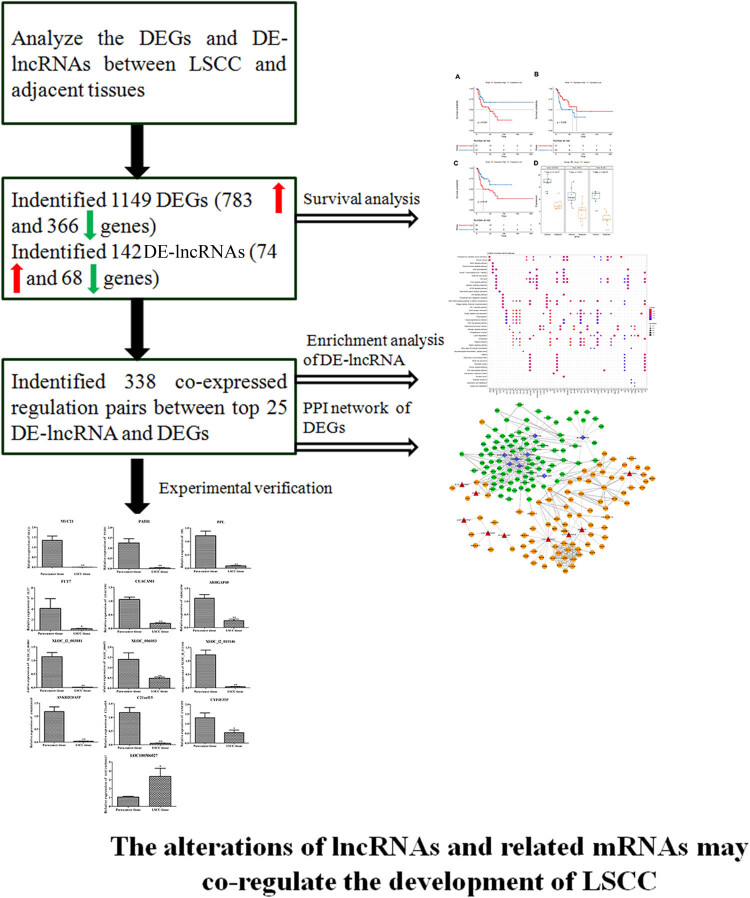

## 1 Introduction

Squamous cell carcinoma of the head and neck is the 6th most common malignancy worldwide with nearly 177,000 new cases in 2018 [1]. Laryngeal squamous cell carcinoma (LSCC) is the second common malignant tumor of the head and neck, comprising 96% of all laryngeal cancers [2]. It has been reported that the mortality rates and crude incidence of laryngeal cancer in China from 2008 to 2012 are 1.01/100,000 and 1.22/100,000, respectively, higher in men than in women [3]. Smoking and alcohol consumption, virus infection, and air pollution are considered as main factors inducing LSCC [4]. Although significant advances in LSCC detection and treatment have been made, the 5-year survival rate and prognosis of LSCC are still poor [5,6]. Thus, it is of great importance to clarify the molecular mechanisms of LSCC to establish more effective biomarkers or appropriate treatment targets.

In the last two decades, the molecular biomarkers and relative regulatory mechanisms of LSCC have been widely investigated [7]. Numerous long noncoding RNAs (lncNRAs) are closely associated with the development of some cancers [8]. For example, lncRNA SNHG1 is overexpressed in LSCC tissues, which is involved in the proliferation and metastasis of LSCC [9]. It is reported that some lncRNAs cooperate with nearby protein coding genes to constitute “lncRNA–mRNA pairs” that affect their function [10]. For instance, Kong et al. [11] indicated that lncRNA FOXC1-FOXCUT pair might be involved in oral squamous cell carcinoma progression. Yang et al. [12] reported that TCONS_00010232, ENST00000564977, and ENST00000420168 might affect CASP3 and FOXQ1 expression in HPV-18 positive cervical cancer cell. Zhou et al. [13] found several lncRNA–mRNA pairs, such as lncRNA-LMO1-2-RIC3 and lncRNA-MCL1-ADAMTSL4, which might play vital roles in the progression of hypopharyngeal squamous cell carcinoma. Besides, Feng et al. [14] suggested that lncRNA NR_027340-ITGB1, lncRNA MIR31HG-HIF1A, and lncRNA SOX2-OT-DDIT4 were important for advanced LSCC. However, the previous studies about lncRNA–mRNA pairs were not enough to elucidate the molecular mechanisms of LSCC development.

In the current study, the lncRNA and mRNA data of GSE84957 and the RNA-seq data of 112 LSCC samples from the cancer genome atlas (TCGA) database were used for the analysis. Differentially expressed genes (DEGs) and differentially expressed lncRNAs (DE-lncRNAs) between LSCC tissues and adjacent normal tissues were identified. Subsequently, co-expression analysis of DEGs and DE-lncRNA was conducted. Protein–protein interaction (PPI) prediction for top 25 DE-lncRNA co-expressed DEGs was performed, followed by Kyoto Encyclopedia of Genes and Genomes (KEGG) pathway enrichment analysis for lncRNA. After that, transcription factor (TF) and microRNA (miRNA) prediction and functional enrichment analysis of co-expressed DEG and survival analysis for key nodes in co-expression network were conducted. Finally, the expressions of several DE-lncRNAs and DEGs in paired samples of LSCC and adjacent tissues were verified using quantitative real-time-PCR (qRT-PCR). We aimed to find significant lncRNA–mRNA pairs and important prognostic genes in the development of LSCC and then tried to elucidate its molecular mechanisms.

## 2 Materials and methods

### 2.1 Data source

The lncRNA and mRNA expression profiles of LSCC were all analyzed in this study. The lncRNA and mRNA dataset GSE84957 involving 9 pairs of primary Stage IV LSCC tissues and adjacent normal tissues were also downloaded from GEO (http://www.ncbi.nlm.nih.gov/geo/) database. The expression data of this dataset were generated from the platform of GPL17843 Agilent-042818 Human lncRNA Microarray 8_24_v2.

In addition, the clinical data and RNA-seq data of 112 LSCC samples were achieved from the cancer genome atlas (TCGA) database. In brief, the clinical data and RNA-seq data of TCGA-head and neck squamous cell carcinoma (TCGA-HNSC) were downloaded from UCSC Genome Browser. According to the clinical information, the samples with tumor location at larynx were selected.

### 2.2 Data preprocessing and identification of DEGs and DE-lncRNAs

After obtaining the raw data of lncRNA–mRNA, the data were preprocessed with linear models for microarray data (limma) software [15], including background correction, data normalization, and concentration prediction. Following data annotation, when several probes were matched to one gene entry, the final expression value was calculated by the mean of these probes. The DEGs analysis between the tumor and control samples was conducted using Bayes test and the *p* values were revised by Benjamini/Hochberg (BH) method. The DEGs and DE-lncRNAs were screened, and |log_2_ fold-change (FC)| > 1 and adjusted *p* value <0.05 were deemed as significantly thresholds. The information of protein coding gene (V32) provided by Gencode (https://www.gencodegenes.org/) database [16] was applied to annotate the RNA-seq data of LSCC samples from TCGA into mRNA and lncRNA expression matrixes for following analysis. Then, the bidirectional hierarchical clustering heatmaps for DEGs and DE-lncRNAs were drawn with pheatmap package (Version 1.0.10, https://cran.r-project.org/web/packages/pheatmap/index.html) in R software [17].

### 2.3 Co-expression analysis of DEGs and DE-lncRNA

The expression matrixes data of DEGs and DE-lncRNAs identified from GSE84957 dataset were extracted to conduct the pearson correlation analysis. The pearson correlation coefficient (*r*) between each DEGs and DE-lncRNA was calculated. Then, DE-lncRNA-DEG pairs with *r* > 0.9 and *p* value <0.05 were selected, among which the pairs of top 25 expression changed DE-lncRNAs and their co-expression DEG was considered as important for following analysis.

### 2.4 Protein–protein interaction (PPI) prediction for top 25 DE-lncRNA co-expressed DEGs

The STRING database (http://string-db.org/) provides the functional partnerships and interactions between proteins for more than 2000 organisms [18]. The PPIs pairs between proteins edited by DEGs from the above significant correlated top 25 DE-lncRNA-DEGs co-expression pairs were analyzed using STRING (version 10.0) with setting PPI score as 0.4. Afterwards, the PPI network construction was conducted using Cytoscape software (version 3.2.0, http://www.cytoscape.org/) [19].

### 2.5 Kyoto Encyclopedia of Genes and Genomes (KEGG) pathway enrichment analysis for lncRNA

KEGG database, as a resource for deciphering genome and pathways, reveals biological interpretation of genes in molecular datasets [20]. lncRNA-enriched pathway was predicted based on functional pathways of each lncRNA co-expressed mRNA using the clusterprofiler package [21] in R software (Version 3.14.0, http://bioconductor.org/packages/3.2/bioc/html/clusterProfiler.html). BH-adjusted *p* value <0.05 and count >1 were used to present the significantly enriched KEGG pathways.

### 2.6 Transcription factor (TF) prediction and functional enrichment analysis for co-expressed DEGs

TFs are major trans-acting factors in transcriptional regulation, which is crucial to investigate the regulatory circuitry underlying complex traits. TRRUST is a database of reference TF-target regulatory interactions in humans based on literature curation, which conducted sentence-based text mining and prioritized the candidate sentences for the cost-effective literature curation [22]. TF was predicted for co-expressed DEGs using TRRUST v2 (https://www.grnpedia.org/trrust/). TF-target genes network was constructed using Cytoscape.

Gee ontology (GO)-biological process (BP), GO-cellular component (CC), GO-molecular function (MF), and KEGG pathway enrichment analyses were performed using R package clusterProfiler v 3.14.0. KEGG pathways and GO terms with adjusted *p* < 0.05 (method: BH) were screened, and the top 10 pathways/terms were presented using bubble chart.

### 2.7 microRNAs (miRNAs) prediction and functional enrichment analysis

miRNAs are noncoding small endogenous RNAs which mediate posttranscriptional gene regulation, which are reported to implicate in various biological processes, such as cell proliferation and apoptosis, disease development, and angiogenesis [23]. Therefore, we further predicted miRNAs for the co-expressed DEGs using Webgestalt (http://www.webgestalt.org/option.php) database. *p* value <0.05 was used to select miRNA-target interactions. miRNAs-target genes network was visualized using Cytoscape. Functional enrichment analyses were performed using R package clusterProfiler v 3.14.0, and BH-adjusted *p* < 0.05 was used to show significant enriched terms.

### 2.8 Survival analysis for key genes in co-expression network

The expression values of all genes and prognosis and survival information were extracted from TCGA database. The genes were divided into low or high expression group based on the median expression in all samples using R package Survival [24] (Version: 2.42-6 https://cran.r-project.org/web/packages/survival/index.html). Genes with *p* < 0.05 in survival analysis were considered as prognosis significantly related genes. In addition, Kaplan–Meier (K–M) survival curves were plotted. Furthermore, clinical information was analyzed based on progress-free survival (PFS) provided by TCGA.

### 2.9 qRT-PCR analysis

In total, five paired LSCC and adjacent nonneoplastic tissues samples were collected from five LSCC patients who underwent surgery in Otolaryngology Department of Gulou Hospital affiliated to Nanjing Medical College, and these tissue samples were then used in this study. The characteristics of patients included in the study are listed in Table A1. Total RNA from frozen tissue (50–100 mg) homogenized in 1 mL TRIZOL reagent was extracted by TRIzol reagent (9109, Takara, Japan) according to the manufacturer’s protocol. Then, qRT-PCR was conducted to verify the expressions of several DEGs and DE-lncRNAs identified in this study. mRNA was reversed transcribed to cDNA using primeScript RT Master MIX (RR036A, Takara), and reverse transcription reaction for miRNA was conducted with PrimeScript II RTase 1st Strand cDNA Synthesis Kit (6210A, Takara). Subsequently, amplification was conducted using Power SYBR Green PCR Master Mix (A25742, Thermo) with the reaction conditions as following: 50°C for 3 min, 95°C for 3 min, and 40 cycles of 95°C for 10 s and 60°C for 30 s. GAPDH was applied as internal controls for mRNAs. Table 1 lists the primer sequences of genes. The 2^−ΔΔCt^ method was applied to calculate relative expression of genes.

**Table 1:** The primer sequences of genes

Gene names	Primer sequences (5′–3′)
CYP4F35P-hF	TCCAGAGCAGGACAAAGAGG
CYP4F35P-hR	AACCACCAAACAGTCAGCAGT
C21orf15-hF	GCCGTGCCCTACAGACC
C21orf15-hR	CTTGATGCCTTAGACCTCCC
ANKRD20A5P-hF	ATGGAAGATCCTGCTGTGAA
ANKRD20A5P-hR	TCCTCTGAAGCCACTGGTAAG
XLOC_006053-hF	CAGCCTGACCATTCCCTT
XLOC_006053-hR	GCAGTCTGGTGGTTCTTATTCTA
XLOC_l2_003881-hF	TGCGTGGCTGCCTCTTA
XLOC_l2_003881-hR	GCATCACTCCTGGGTGTCTT
XLOC_l2_011146-hF	GTCTTCCTGAAGCCACACAGA
XLOC_l2_011146-hR	TCCTCCAGAGTCTCCCATTAAA
LOC100506027-hF	ACAGCGATACCAGGCAGAC
LOC100506027-hR	GCATTCGTGGCGATAAGG
MUC21-hF	GAATGCACACAACTTCCCATAGT
MUC21-hR	GGCTATCGAGGATACTGGTCTC
CEACAM1-hF	GATCCTATACCTGCCACGCC
CEACAM1-hR	CCTGTGACTGTGGTCTTGCT
FUT7-hF	CACCTGAGTGCCAACCGAA
FUT7-hR	CACCCAGTTGAAGATGCCTCG
PADI1-hF	TGCAGACATGGTCGTATCTGT
PADI1-hR	GCCCAGAGCTTGGTCTTCC
PPL-hF	CCGGAGCATCTCTAACAAGGA
PPL-hR	GCATCCGCCTCTAGCACAT
ARHGAP40-hF	AGCCTTCAACATGGACTCTGC
ARHGAP40-hR	TTTGGGGACGGTAAACTTCGG
GAPDH-hF	TGACAACTTTGGTATCGTGGAAGG
GAPDH-hR	AGGCAGGGATGATGTTCTGGAGAG


**Informed consent:** Informed consent has been obtained from all individuals included in this study.
**Ethical approval:** The research related to human use has been complied with all the relevant national regulations, institutional policies, and in accordance with the tenets of the Helsinki Declaration, and has been approved by the Ethics Committee of the Gulou Hospital affiliated to Nanjing Medical College.

### 2.10 Statistics analysis

All the data were presented as mean ± standard deviation. Statistics analysis was performed using Graphpad prism 5 (Graphpad Software, San Diego, CA), and the express values between groups were compared using Student’s *t*-test. *p* < 0.05 was deemed statistically significant.

## 3 Results

### 3.1 Identification of DEGs and DE-lncRNAs

**Figure 1:**
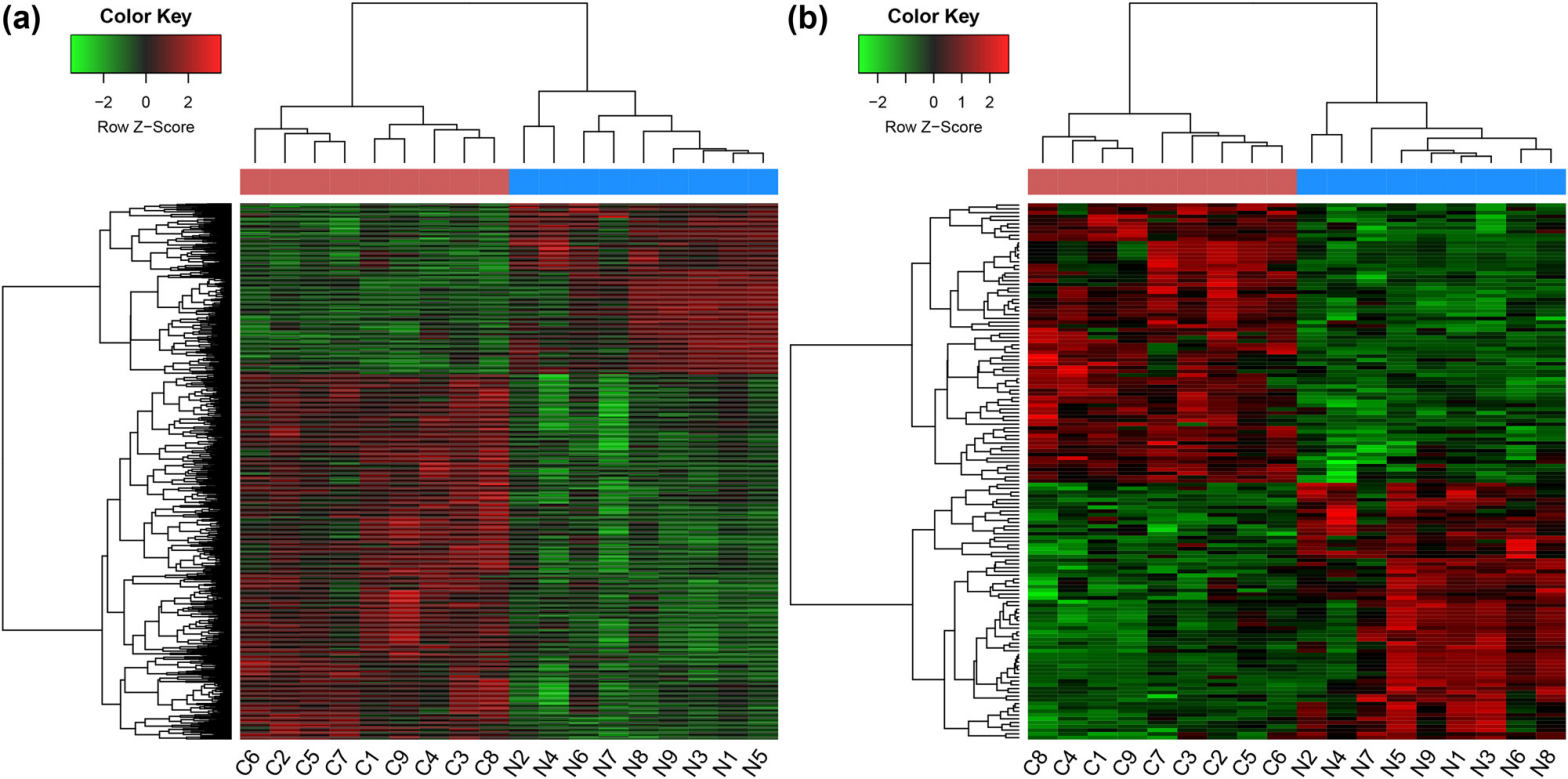
Heat maps of DEGs (a) and DE-lncRNA (b) in LSCC. *X*-axis shows the samples, and the *Y*-axis shows the DEGs or DE-lncRNA. DEGs: differentially expressed genes; DE-lncRNAs: differentially expressed lncRNAs; LSCC: laryngeal squamous cell carcinoma.

Under the cut-off of |log_2_ FC| > 1 and adjusted *p* value <0.05, a total of 1,149 DEGs (including 783 up- and 366 downregulated DEGs) and 142 DE-lncRNAs (including 74 up- and 68 downregulated DE-lncRNAs) were identified across LSCC tissues and normal tissues samples. The results of heatmaps showed that these DEGs and DE-lncRNAs could clearly distinguish the LSCC samples from normal samples, which verified DEGs and DE-lncRNAs were credible and could be used for following analysis (Figure 1).

### 3.2 Co-expression analysis of top 25 DE-lncRNA and DEGs

**Figure 2:**
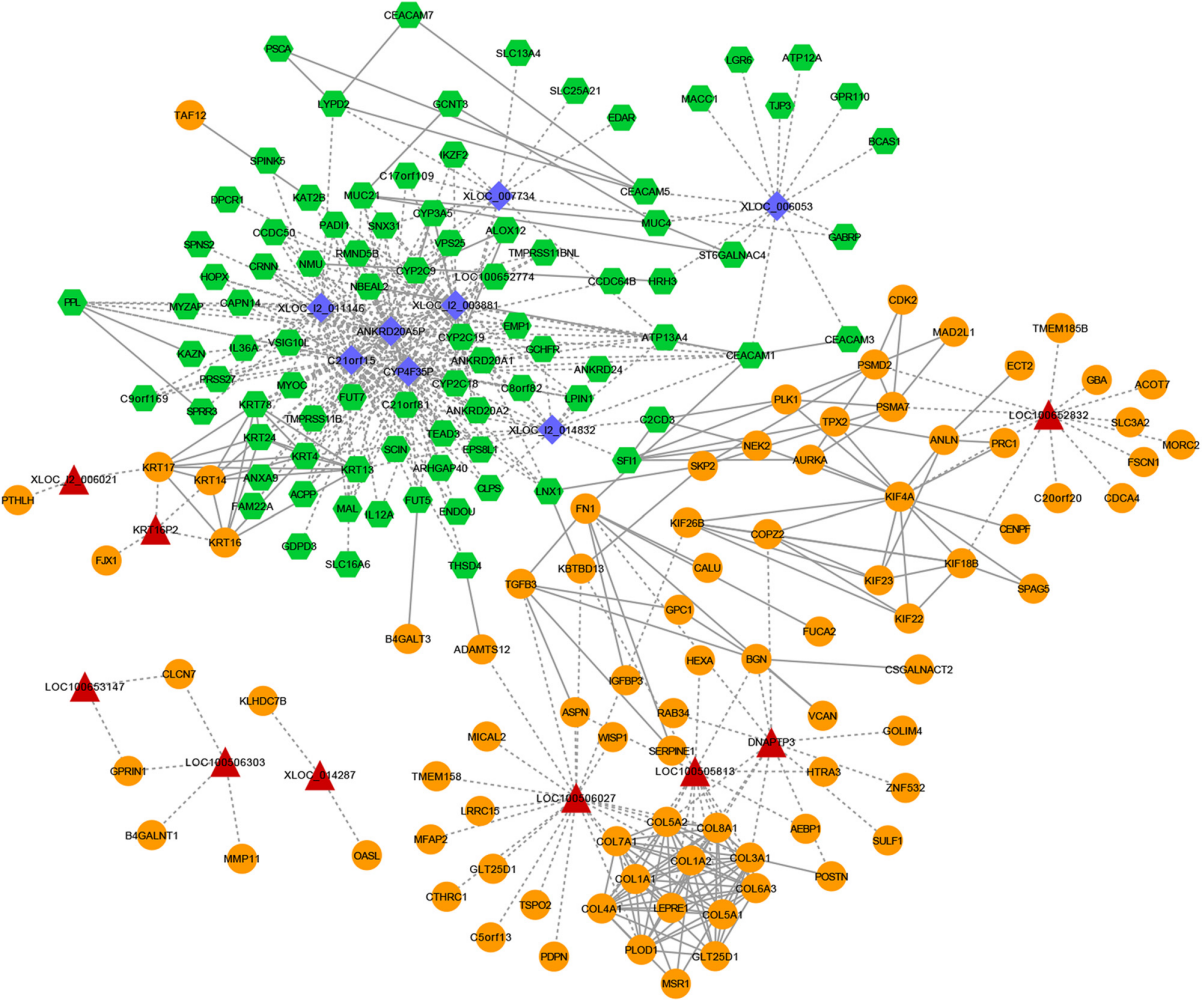
The lncRNA–mRNA co-expression network. Purple diamond: downregulated lncRNA; red triangle: upregulated lncRNA; green hexagon: downregulated mRNA; orange circle: upregulated mRNA; dotted line: lncRNA–mRNA co-expression pairs; solid line: protein–protein interaction (PPI) pairs.

According to the given threshold, a total of 338 co-expressed regulation pairs between top 25 DE-lncRNA and DEGs (involving 17 DE-lncRNA and 145 DEGs) were identified. PPI prediction was performed for these 145 DEGs, of which 174 interaction pairs were predicted for 82 DEGs. Then, lncRNA–mRNA network (Figure 2, Table S1) was constructed by integrating these relations. It showed that seven significant downregulated DE-lncRNAs with lowest log_2_ FC values (ANKRD20A5P, C21orf15, CYP4F35P, XLOC_I2_011146, XLOC_006053, and XLOC_I2_003881) and one of top 3 upregulated LOC100506027 were highlighted in network. Furthermore, some DEGs were co-expressed with these lncRNA, such as FUT7, PADI1, PPL, ARHGAP40, MUC21, and CEACAM1.

### 3.3 KEGG pathway enrichment analysis for DE-lncRNA

**Figure A1:**
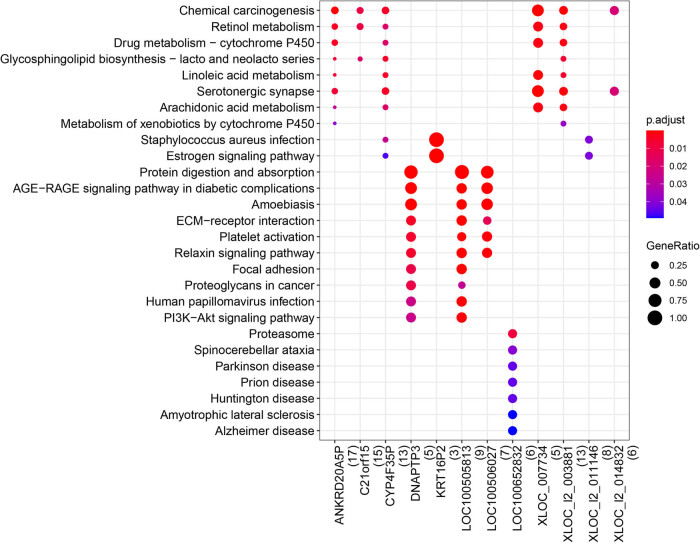
Significantly enriched Kyoto encyclopedia of genes and genomes (KEGG) pathway for lncRNAs in lncRNA–mRNA network. Abscissa: lncRNA; Ordinate: enriched KEGG pathways; point size: GeneRatio, color shift from blue to red indicates *p* adjust value from low to high.

KEGG pathway enrichment analysis for lncRNAs in the lncRNA–mRNA network was performed based on each lncRNA co-expressed mRNA (Figure A1). It could be seen that there were similarities and differences on the involved KEGG pathways of these lncRNAs. For example, XLOC_l2_003881 and XLOC_007734 were significantly related to chemical carcinogenesis, drug metabolism-cytochrome P450 and serotonergic synapse, etc. While LOC100505813 and DNAPTP3 were associated with ECM−receptor interaction, platelet activation, and focal adhesion, LOC100652832 was implicated in proteasome.

### 3.4 TF prediction and functional enrichment analysis for co-expressed DEGs

**Figure 3:**
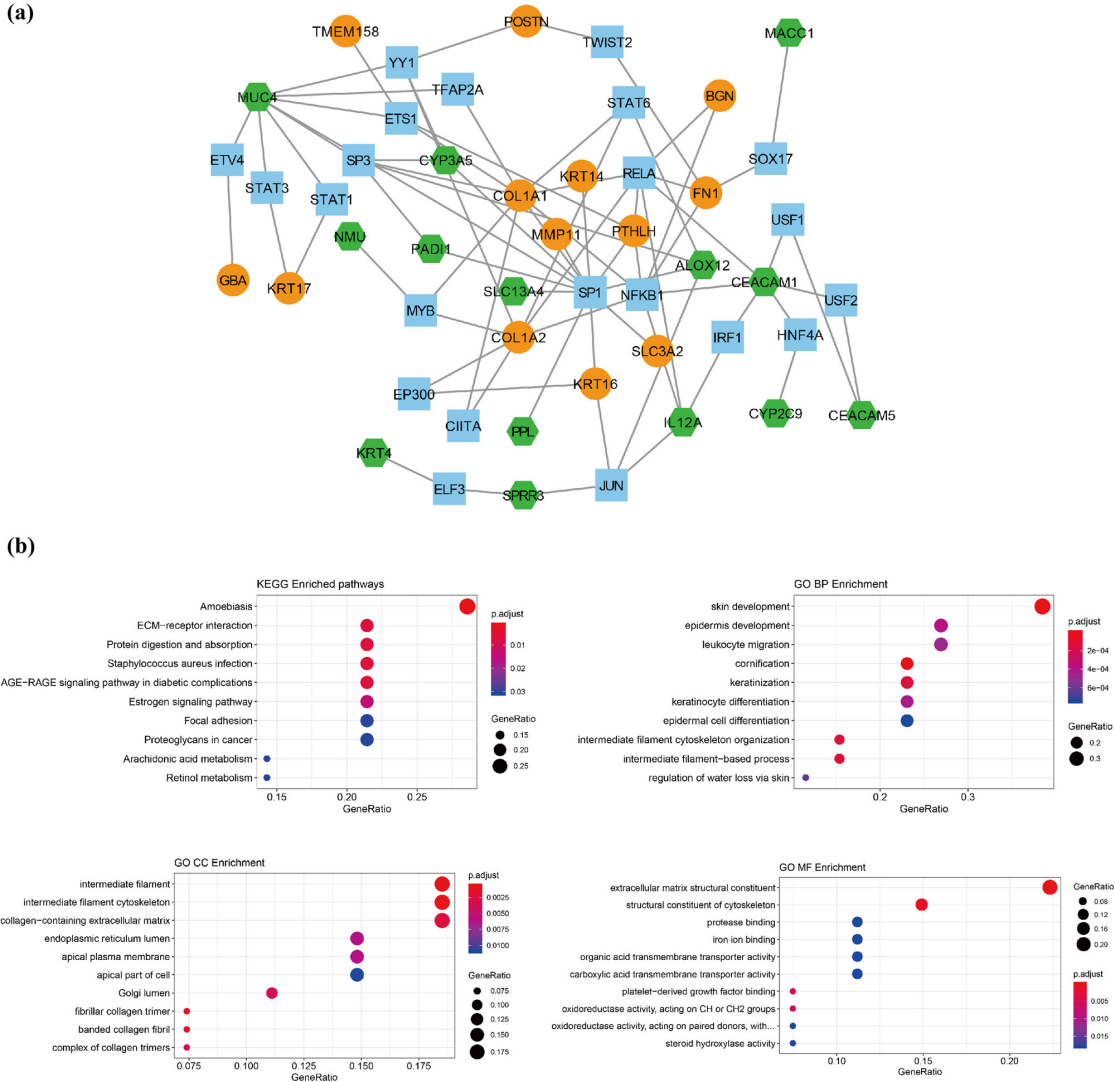
Transcription factor (TF) prediction and functional enrichment. (a) The TF-mRNA network. Blue square: TFs; orange circle: upregulated mRNA; green hexagon: downregulated mRNA. (b) The top 10 gene ontology (GO) terms and Kyoto Encyclopedia of Genes and Genomes (KEGG) pathways enriched by differentially expressed genes (DEGs) in TF-mRNA network. Point size: GeneRatio, color shift from blue to red indicates *p* adjust value from low to high.

After TF prediction for 145 DEGs, 75 TF-mRNA pairs were obtained, which included 22 TFs (e.g., SP1, NFKB1, RELA, and JUN) and 27 DEGs (e.g., upregulated COL1A1, MMP11, PTHLH, and KRT14; downregulated PPL and CEACAM1) (Figure 3a).

As presented in Figure 3b, DEGs in TF-mRNA network were markedly enriched in amoebiasis, ECM−receptor interaction, protein digestion and absorption, staphylococcus aureus infection, and AGE-RAGE signaling pathway in diabetic complications. Furthermore, the evidently enriched GO-BP terms included skin development, cornification, and keratinization; GO-CC terms included intermediate filament, intermediate filament cytoskeleton, and collagen-containing extracellular matrix; while GO-MF terms included extracellular matrix structural constituent and structural constituent of cytoskeleton.

### 3.5 miRNAs prediction and functional enrichment analysis

**Figure 4:**
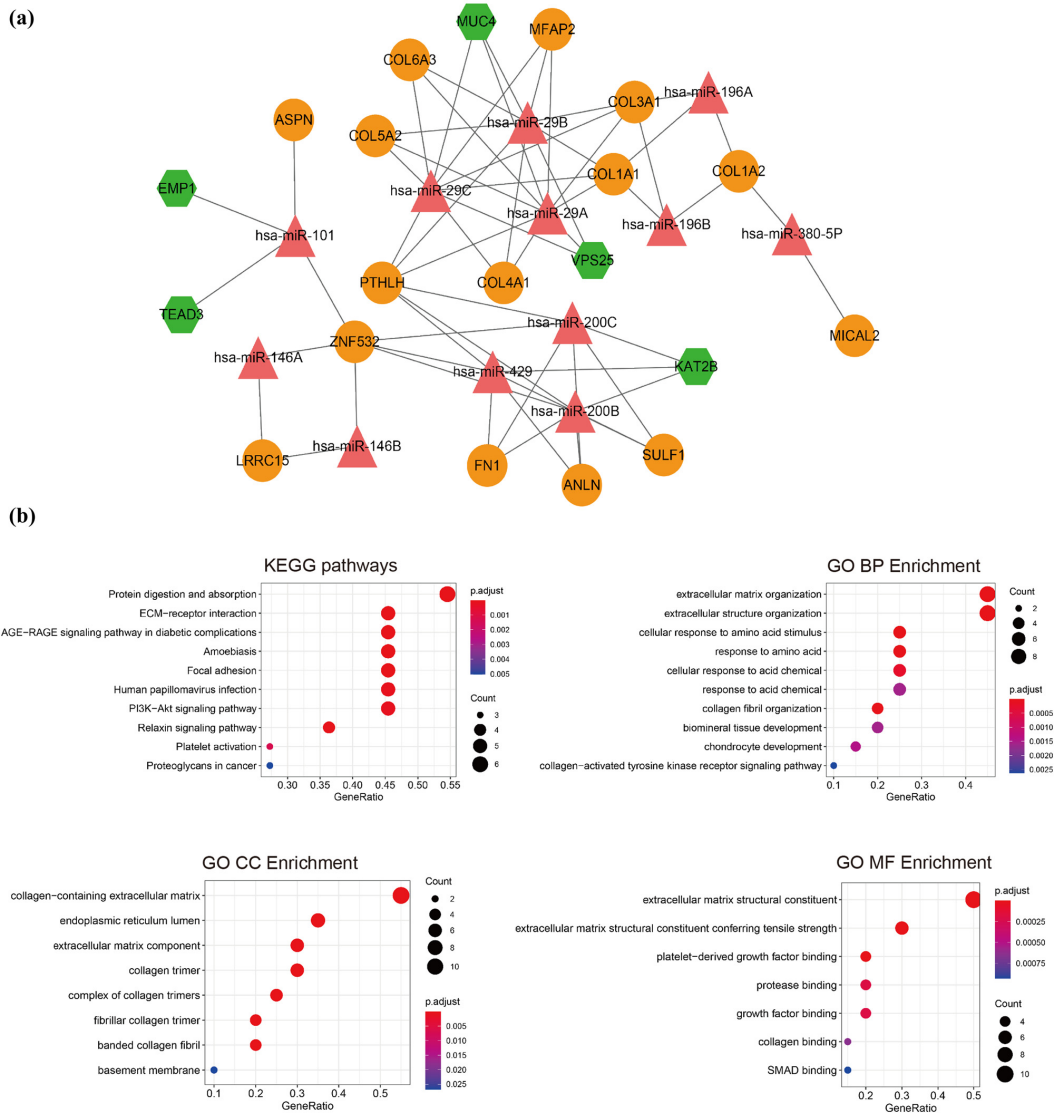
microRNAs (miRNAs) prediction and functional enrichment. (a) The miRNA-target network. Green hexagon: downregulated mRNAs; orange circle: upregulated mRNA; red triangle: miRNAs. (b) The top 10 gene ontology (GO) terms and Kyoto Encyclopedia of Genes and Genomes (KEGG) pathways enriched by differentially expressed genes (DEGs) in miRNA-target network. Point size: GeneRatio, color shift from blue to red indicates *p* adjust value from low to high.

Following miRNAs prediction for 145 DEGs, the miRNA-target network was constructed (Figure 4a). The miRNA-target network contained 12 miRNAs (e.g., miR-200b/c, miR-29a/b/c and miR-429) and 20 DEGs (e.g., upregulated COL1A1, PTHLH, COL4A1; downregulated MUC4 and KAT2B).

Similarly, DEGs in miRNA-target network were significantly enriched in ECM−receptor interaction, focal adhesion, and PI3K − Akt signaling pathways. The enriched GO-BP contained extracellular matrix/structure organization, cellular response to amino acid/acid chemical; GO-CC terms included collagen − containing extracellular matrix and endoplasmic reticulum lumen; and GO-MF terms included extracellular matrix structural constituent and platelet − derived growth factor binding (Figure 4b).

### 3.6 Survival analysis

**Figure 5:**
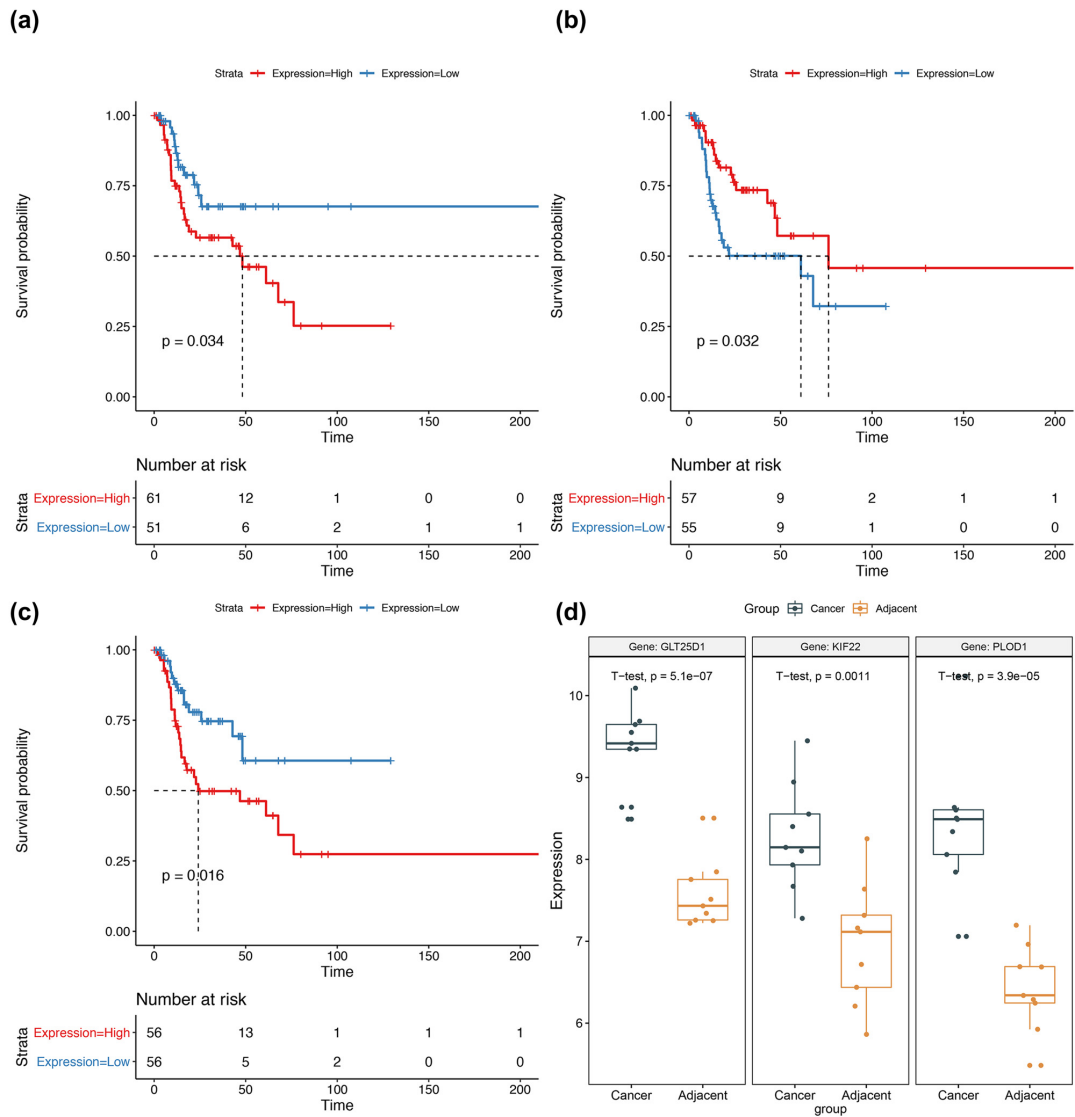
Survival analyses for GLT25D1 (a), KIF22 (b), and PLOD1 (c), and the box plot for the expression values of these three genes in GSE84957 (d).

Survival analyses were conducted for one lncRNA (HCG22) and all the above mRNA nodes. The results showed that PLOD1 (*p* = 0.016), GLT25D1 (also named COLGALT1, *p* = 0.034), and KIF22 (*p* = 0.032) were significantly associated with prognosis (Figure 5a–c). The expression values of these three genes in GSE84957 were presented as box plot (Figure 5d).

### 3.7 Verification of gene expressions

**Figure 6:**
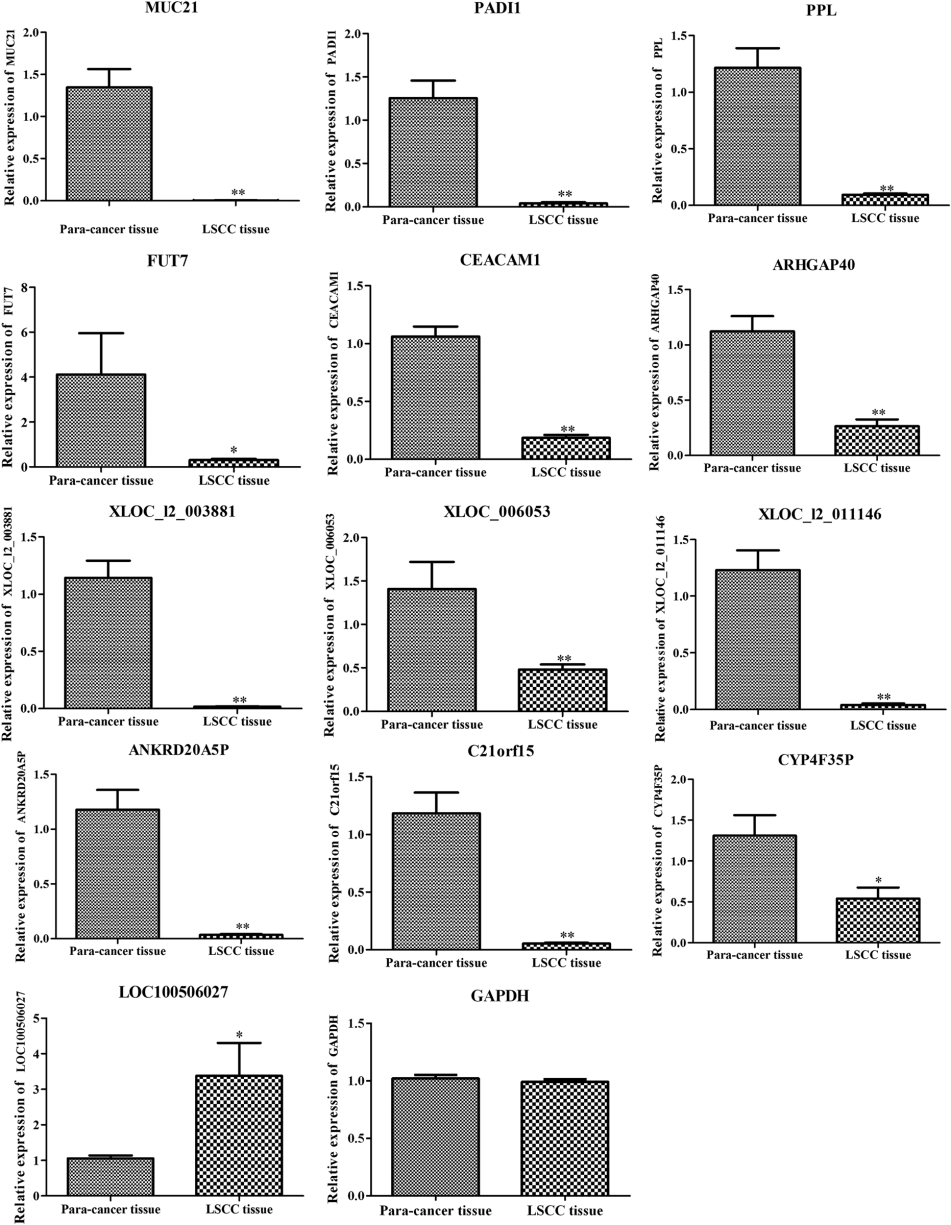
Relative mRNA expressions of MUC21, PADI1, PPL, FUT7, CEACAM1, ARHGAP40, XLOC_I2_003881, XLOC_006053, XLOC_I2_011146, ANKRD20A5P, C21orf15, CYP4F35P, LOC100506027, and GAPDH in LSCC tissues compared with adjacent tissues detected by real-time quantitative polymerase chain reaction. ** represents *p* < 0.01, and * represents *p* < 0.005 between LSCC and adjacent tissues samples.

ANKRD20A5P, C21orf15, CYP4F35P, XLOC_I2_011146, XLOC_006053, XLOC_I2_003881, and LOC100506027 with larger |log_2_ FC| were co-expressed with more DEGs in lncRNA–mRNA network, thus the expression of these 7 lncRNA was verified. Furthermore, each of FUT7, PADI1, PPL, ARHGAP40, MUC21, and CEACAM1 was co-expressed with several of the above 7 lncRNAs, thus these 6 genes were verified. The qRT-PCR results suggested that the expressions of MUC21, CEACAM1, FUT7, PADI1, PPL, ARHGAP40, ANKRD20A5P, C21orf15, CYP4F35P, XLOC_I2_003881, XLOC_I2_011146, and XLOC_006053 were downregulated in LSCC compared with that in adjacent tissues. The expression of LOC100506027 was upregulated in LSCC compared with that in adjacent tissues (Figure 6).

## 4 Discussion

In the current study, lncRNA and mRNA expression profiles of LSCC were comprehensively analyzed to find significant lncRNA–mRNA pairs and important prognostic genes for LSCC. The lncRNA–mRNA network showed that top downregulated ANKRD20A5P, C21orf15, CYP4F35P, XLOC_I2_011146, XLOC_006053, and XLOC_I2_003881 and one of top 3 upregulated LOC100506027 were highlighted in network. Furthermore, some DEGs, such as FUT7, PADI1, PPL, ARHGAP40, MUC21, and CEACAM1, were co-expressed with these above lncRNAs. Survival analysis showed that PLOD1, GLT25D1 (COLGALT1), and KIF22 were significantly associated with prognosis of LSCC. In addition, the qRT-PCR results suggested that the expressions of MUC21, CEACAM1, FUT7, PADI1, PPL, ARHGAP40, ANKRD20A5P, C21orf15, CYP4F35P, XLOC_I2_003881, XLOC_I2_011146, and XLOC_006053 were significantly downregulated, whereas the expression of LOC100506027 was significantly upregulated in LSCC tissues compared with that in para-cancer tissues.

It was reported that PLOD1 is a potential prognostic marker in gastrointestinal cancer [25]. Yamada et al. [26] suggested that aberrant expressed PLOD1 was related to pathogenesis of bladder cancer, and it might be a potential prognostic marker for this cancer. PLOD1 can promote cell migration and growth in osteosarcoma [27]. Suppression of KIF22 inhibits cancer cell proliferation through delaying mitotic exit [28]. Zhang et al. [29] indicated that KIF22 was associated with clinical outcome and tumor progression in prostate cancer. KIF22 is involved in the migration and proliferation of gastric cancer cells through MAPK-ERK pathways [30]. As previously reported, COLGALT1 is involved in the progression of mammary tumor metastases [31]. Wang et al. [32] indicated that COLGALT2 played role in the proliferation of osteosarcoma. Not too much previous studies reported the roles of these three genes in LSCC. Combined with our present survival analysis results, we inferred that PLOD1, GLT25D1 (COLGALT1), and KIF22 might be potential prognostic markers for LSCC development.

Our qRT-PCR results showed that the expression of MUC21, CEACAM1, FUT7, PADI1, PPL, and ARHGAP40 was downregulated in LSCC tissues compared with that in para-cancer tissues. MUC21, as a member of the mucin family, may play a protective role against external stimuli in mucus layer on mucosal surfaces [33]. There is growing evidence that mucin families are responsible for epithelial carcinomas, especially LSCC [33]. Yuan et al. have reported that MUC21 is associated with differentiation and carcinogenesis of squamous epithelial di [34]. Nair et al. have predicted the downregulation of MUC21 in LSCC tumors via gene expression profile analysis [35], which is consistent with our result. Some studies showed that CEACAM1 played roles in tumorigenesis. The loss of expression and genetic alteration of the CEACAM1 may be an early event for colorectal cancers development [36]. CEACAM1 is related to oral tumors progression [37]. Importantly, Lucarini et al. [38] demonstrated that CEACAM1 was involved in LSCC progression and might be a potential therapeutic target for LSCC. There were no researches about the roles of FUT7, PADI1, PPL, and ARHGAP40 in LSCC, but the roles of these genes or the related genes in other cancers were reported. For example, lower expression of PPL is related to cancer-specific survival and pathological stage in urothelial carcinoma of the urinary bladder [39]. Cui et al. [40] demonstrated that overexpression of exogenous FUT7 contributed to migration and adhesion of cell line MDAMB-231 of breast cancer. PADI2 inhibits proliferation of colon cancer cells [41] and can be used as a potential marker for breast cancer [42]. Downregulated ARHGAP10 inhibits tumorigenicity of ovarian cancer cells [43]. ARHGAP17 plays tumor suppressive role in colon cancer via Wnt/β-Catenin Signaling [44]. Thus, MUC21, CEACAM1, FUT7, PADI1, PPL, and ARHGAP40 may be associated with the development of LSCC.

Chromosome 21 open reading frame 15 (C21orf15) is a lncRNA located in the juxtacentromeric region of human chromosome 21 with domain of spliced expressed sequence tags AJ003450 [45]. It has been reported that C21orf15 is predicted to be upregulated in metastatic prostate cancer [46], whereas our RT-PCR result showed that C21orf15 was downregulated in LSCC tissue. However, few studies reported the function of C21orf15. Combined with our present study that C21orf15 was co-expressed with MUC21, CEACAM1, FUT7, PADI1, PPL, and ARHGAP40, we inferred that C21orf15-MUC21/CEACAM1/FUT7/PADI1/PPL/ARHGAP40 were lncRNA–mRNA pairs that were involved in LSCC development. That is to say, C21orf15 may affect LSCC development by modulating the expression of MUC21/CEACAM1/FUT7/PADI1/PPL/ARHGAP40. Lastly, there are no previous researches that studied the functions of ANKRD20A5P, CYP4F35P, XLOC_I2_003881, XLOC_I2_011146, XLOC_006053, and LOC100506027. Further researches are needed to clarify the function of these lncRNA in LSCC. Besides, the co-expression relationships of 7 lncRNAs and these genes were needed to be verified by experiments in future.

## 5 Conclusion

In summary, PLOD1, GLT25D1, and KIF22 may be potential prognostic markers for LSCC development. MUC21, CEACAM1, FUT7, PADI1, PPL, and ARHGAP40 may be involved in the development of LSCC. C21orf15-MUC21/CEACAM1/FUT7/PADI1/PPL/ARHGAP40 are important lncRNA–mRNA pairs that play significant roles in LSCC. ANKRD20A5P, CYP4F35P, XLOC_I2_003881, XLOC_I2_011146, XLOC_006053, and LOC100506027 may be vital lncRNAs in LSCC progression. These lncRNAs and related mRNAs may be used for potential therapeutic targets of LSCC.

## Appendix

**Table A1:** The characteristics of the patients from Gulou hospital

Patients	Diagnosis	Surgery	Age (years)	Sex	Smoking	Drinking	Tumor size	TNM stage	Lymph node metastasis
P1	LSCC, dyspnea	Total laryngectomy and bilateral neck dissection	45	Male	Yes, smoking for 30 years	Yes	3.5 cm × 2 cm × 1 cm	IVA (T4aN2bcM0)	N2b
P2	LSCC, dyspnea	Total laryngectomy and bilateral neck dissection	63	Male	Yes, smoking for 40 years	No	3 cm × 2.5 cm × 1 cm	IVA (T4aN2bcM0)	N1
P3	LSCC	Total laryngectomy and bilateral neck dissection	79	Male	Yes, smoking for 60 years	No	3 cm × 2 cm × 0.6 cm	IVA (T4aN2bcM0)	N0
P4	LSCC	Total laryngectomy and left neck dissection	64	Male	Yes, smoking for 30 years	Yes	3.5 cm × 3 cm × 1.5 cm	IVA (T4aN2bcM0)	N0
P5	LSCC	Total laryngectomy and right neck dissection	70	Male	Yes, smoking for 50 years	Yes	2.5 cm × 1.4 cm × 1 cm	III (T3N0cM0)	N0
